# Fabricating Na/In/C Composite Anode with Natrophilic Na–In Alloy Enables Superior Na Ion Deposition in the EC/PC Electrolyte

**DOI:** 10.1007/s40820-021-00756-7

**Published:** 2021-12-09

**Authors:** Hui Wang, Yan Wu, Ye Wang, Tingting Xu, Dezhi Kong, Yang Jiang, Di Wu, Yongbing Tang, Xinjian Li, Chun-Sing Lee

**Affiliations:** 1 Key Laboratory of Material Physics of Ministry of Education, School of Physics and Microelectronics, Zhengzhou University, Zhengzhou, 450052 People’s Republic of China; 2 Department of Chemistry, Center of Super-Diamond and Advanced Films (COSDAF), City University of Hong Kong, Hong Kong SAR, 999077 People’s Republic of China; 3 School of Materials Science and Engineering, Hefei University of Technology, Hefei, 230009 Anhui People’s Republic of China; 4 Shenzhen Institutes of Advanced Technology, Chinese Academy of Sciences, Shenzhen, 518055 People’s Republic of China

**Keywords:** EC/PC electrolyte, Sodium metal batteries, Na/In/C electrode, Dendrite-free and smooth morphology, Theoretical simulations

## Abstract

In conventional ethylene carbonate (EC)/propylene carbonate (PC) electrolyte, sodium metal reacts spontaneously and deleteriously with solvent molecules. This significantly limits the practical feasibility of high-voltage sodium metal batteries based on Na metal chemistry. Herein, we present a sodium metal alloy strategy via introducing NaIn and Na_2_In phases in a Na/In/C composite, aiming at boosting Na ion deposition stability in the common EC/PC electrolyte. Symmetric cells with Na/In/C electrodes achieve an impressive long-term cycling capability at 1 mA cm^−2^ (> 870 h) and 5 mA cm^−2^ (> 560 h), respectively, with a capacity of 1 mAh cm^−2^. In situ optical microscopy clearly unravels a stable Na ion dynamic deposition process on the Na/In/C composite electrode surface, attributing to a dendrite-free and smooth morphology. Furthermore, theoretical simulations reveal intrinsic mechanism for the reversible Na ion deposition behavior with the composite Na/In/C electrode. Upon pairing with a high-voltage NaVPOF cathode, Na/In/C anode illustrates a better suitability in SMBs. This work promises an alternative alloying strategy for enhancing Na metal interfacial stability in the common EC/PC electrolyte for their future applications.

**Supplementary Information:**

The online version contains supplementary material available at 10.1007/s40820-021-00756-7.

## Highlights

- Na/In/C composite anode tailored with natrophilic NaIn and Na_2_In phases was constructed via a facile molten metal immersion method.
- Long-term Na ion plating/stripping stability in the common ethylene carbonate/propylene carbonate electrolyte was achieved with Na/In/C anode.
- Na ion deposition reversibility of Na/In/C anode was analyzed by in situ microscope, ab initial molecular dynamics and finite element simulations.

**Supplementary Information:**

The online version contains supplementary material available at 10.1007/s40820-021-00756-7.

## Graphical abstract

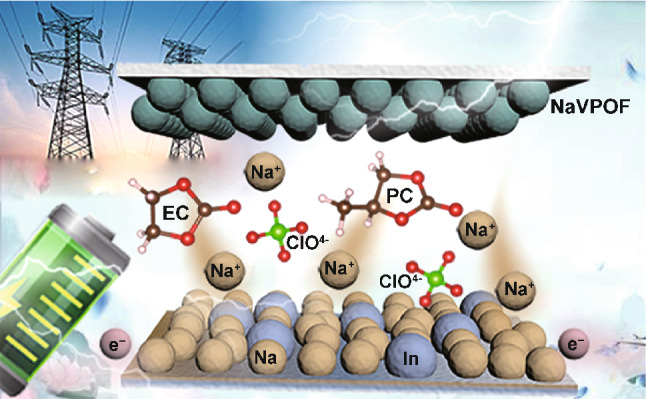

## Introduction

Beyond traditional intercalation chemistry, advanced lithium metal batteries (LMBs) systems, such as Li–S [1–4] and Li-O_2_ [5, 6], have dramatically boosted the energy density of lithium-ion batteries. Nevertheless, high cost and scarcity of metallic lithium largely stifle the feasibility of LMBs [7, 8]. Comparatively, by virtue of materials abundance, competitive theoretical capacity (1166 mAh g^−1^) and desirable redox potential (− 2.71 V vs. the standard hydrogen potential), sodium metal batteries (SMBs) provide an appealing alternative for LMBs [9–11, 11]. As a result, this has prompted burgeoning research interest on SMBs, such as Na–S/Se/Te [12–19], Na–O_2_ [19–21] and Na–P [22–24] systems.

Albeit with these collective attributes, the deployment of Na metal still confronts huge challenges including catastrophically safety issues and poor cycling reversibility. First, owing to its lower Lewis acidity and larger atomic radius, Na metal reacts much more severely with flammable organic liquid electrolytes than Li metal [25, 26]. Second, non-uniform Na ion platting and stripping often lead to electrochemical and/or morphological instabilities [27–33]. All of these problems would cause various unwanted consequences such as electrolyte depletion, detrimental Na dendritic growth, large volume variation, inferior Coulombic efficiency (CE), poor quality SEI (solid electrolyte interphase) and ultimately cells’ failure. Thus far, unremitting efforts have been dedicated to restrict Na dendritic proliferation and improve long-term cyclability. These include modifying molecular structure of electrolytes [34], fabricating protective surface coating [35, 36], building steady artificial SEI [37], synthesizing novel Na ion deposition substrates [38, 39], innovating liquid Na–K alloy [30] and designing robust solid-state electrolytes (SSEs) [40–42]. These rational approaches undoubtedly enhance Na ion deposition stability, while challenge still remains regarding to Na dendrite issues and fabrication complexity. Particularly, (i) ether-based electrolytes can easily decompose at potentials above ~ 3.8 V for SIBs (above ~ 4.0 V for lithium-ion batteries) and thus would compromise cells’ working voltages and energy densities [43]; (ii) unsatisfactory interfacial compatibility between Na metal and SSEs as well as Na dendritic growth within the SSEs necessitate further enhancements [40]. In comparison, by virtue of antioxidant capability, easy tunability and low cost, EC/PC electrolyte has better prospects for fabricating high-voltage SMBs. Nevertheless, continuous parasitic reactions between aggressive Na metal and EC/PC electrolyte still occur regardless of artificial SEI, surface coating and additives [44]. In this sense, tailoring Na metal anode to be more compatible with ester-based electrolytes is strongly desirable for the implementation of viable SMBs with high operating potentials and energy densities.

In this manuscript, we prepare a Na/In/C composite to further stabilize Na metal anode in the EC/PC electrolyte. In doing so, symmetric cells with Na/In/C electrodes can yield superior operational life spans at 1 (> 870 h), 2 (> 710 h) and 5 (> 560 h) mA cm^−2^, respectively, with a capacity of 1 mAh cm^−2^. Stable Na ion deposition behavior is also evidenced at a higher capacity of 3 mAh cm^−2^. Scanning electron microscopy (SEM) and in situ optical microscopy results clearly demonstrate that Na ions smoothly deposited upon the Na/In/C surface without any sodium dendrites. On the contrary, porous and dendritic morphology is observed on a pure Na metal surface after 5 min deposition at 1.0 mA cm^−2^. Ab initial molecular dynamics (AIMD) simulation, density functional theory (DFT), finite element (FE) calculations and electrochemical analysis clearly unveil that the presence of In promotes Na ions nucleation dynamics and deposition stability. Moreover, when paired with a high-voltage Na_3_V_2_O_2_(PO_4_)_3_ cathode (up to 4.3 V), the sodium metal full cells utilizing Na/In/C anode demonstrate a much more promising capacity retention upon extensive cycles. All of these comprehensive results establish a deep insight into the construction of Na/In/C composite anode to improve the cycling stability of SMBs.

## Experimental Section

### Fabrication of Na/In/C Composite Electrode

Typically, a piece of carbon cloth was initially cut into circular disks of ~ 1 cm diameter via a punch machine. Then, these disks were totally immersed into ethanol with an ultrasonication treatment for 3 h. Next, the plates were dried at 80 °C under vacuum for 24 h. Subsequently, metal Na foil, metallic In powder and carbon cloth disks (weight ratio of Na:In:C = 10:1:4) were put into a nickel crucible maintaining at ~ 400 °C for 10 min. Finally, liquefied Na and In can thoroughly infuse into the entire carbon cloth plate, creating the Na/In/C composite. Average weight of one Na/In/C composite disk (0.785 cm^−2^) is 9.3 mg, and the mass loading of Na within the Na/In/C composite is 7.9 mg cm^−2^.

### ***Synthesis of Na***_***3***_***V***_***2***_***O***_***2***_***(PO***_***4***_***)***_***2***_***F (NaVPOF)***

Briefly, 0.1 g hexadecyl trimethyl ammonium bromide (CTAB) were firstly added into 36 mL DMF and sonicated for 20 min. Secondly, a solution comprising of 0.0015 mol NaF, 0.001 mol NH_4_VO_3_ and 8 mL deionized water was gradually drop-wisely into DMF. Then, 8 mL (NH_4_)_2_HPO_4_ (0.001 mol) aqueous solution was mixed with the above solution to guarantee the required stoichiometric ratio (Na:V:P:F = 3:2:2:3). Finally, this solution was sealed into the 60-mL Teflon autoclaves and retained at 180 ℃ for 10 h.

### Materials Characterization

X-ray diffractometer (XRD) patterns of Na, Na/In/C and NaVPOF were obtained with an X-ray diffractometer utilizing Cu-K radiation. A QUATTRO S scanning electron microscope was employed to obtain the morphology and microstructure of the cycled Na and Na/In/C electrodes. X-ray photoelectron spectroscopy (XPS, VG ESCALAB 220i-XL) was carried out to investigate surface chemical properties of the cycled Na and Na/In/C electrodes. Real-time Na ion dynamics deposition and surface morphology evolution process on the Na and Na/In/C electrode were studied by in situ optical microscopy.

### Electrochemical Measurements

To assess the Na ion stripping/plating stability, symmetrical Na||Na and Na/In/C||Na/In/C cells were assembled in a glove box. Electrochemical deposition curves and long-term cycling life span of these cells were tested with a MACCOR battery cycler at 1, 2 and 5 mA cm^−2^ with a capacity of 1 and 3 mAh cm^−2^. As to the full-cell testing, NaVPOF, carbon black and polyvinylidene fluoride with a weight ratio of 8:1:1 were leveraged as active material, conductive additive and binder, respectively. 1.0 M NaClO_4_ in EC/PC (1:1 volume ratio) with 5 wt% fluoroethylene carbonate (FEC) was adopted as electrolyte. Mass loading of the NaVPOF cathode/pure Na foil anode is 14.2/7.9 mg cm^−2^. Cyclic voltammetry (CV) measurements were taken with a Zahner electrochemical workstation (voltage range of 2.5–4.3 V, 0.5 mV s^−1^ scan rate) to study the Na ion deposition kinetics and redox reversibility. Rate performance and long-term cycling stability tests were done from 0.05 C to 5 C rates. Interfacial electrochemical impedance spectroscopy (EIS) features were determined over a frequency range from 1 MHz to 100 mHz through an impedance analyzer (Zennium pro).

### Computational Details

DFT simulations were firstly conducted to determine Na atom adsorption sites, binding energies and charge differential density upon (100), (110) and (111) crystal planes of Na metal and NaIn as well as (311) crystal plane of Na_2_In. All the DFT calculations were coped with projector augmented plane wave method [45]. The exchange–correlation interaction among itinerant electrons is handled by the Perdew–Burke–Ernzerhof (PBE) functional [46]. van der Waals interactions between Na atom and Na metal or NaIn/Na_2_In surface were treated with semiempirical London dispersion corrections of Grimme et al. [47]. Cutoff energy of the plane wave basis was set at 600 eV to ensure strict force and energy convergence criterion, and the k-point mesh (Monkhorst–Pack) was 7 × 7 × 1. AIMD simulations were then performed to clarify dynamic Na atoms movement process on the (011) crystal surface of Na metal and NaIn. A lattice matching interface consisting of six layers of NaIn and three layers of Na metal were fabricated. All the AIMD simulations were done with Gaussians and plane wave (GPW) method implemented in the CP2K software package with a single Γ point and the energy cutoff was set at 300 Ry [48]. DFT-D3 and PBE were employed to deal with long-range weak van der Waals and electron exchange–correlation interactions. All the configurations were allowed to relax for 1.2 ps (time step is 0.5 fs) at 300 K in the canonical ensemble (NVT) dictated by Nose–Hoover thermostat. Morphology evolution of Na metal surface with distinct overpotential and bump shape was further investigated using finite element simulation (COMSOL Multiphysics) utilizing tertiary current distribution and deformed geometry modules. The Na ion deposition kinetics at the Na or Na/In surface were subjected to the concentration-dependent Butler–Volmer equation:

1 $$i{\text{ }} = {\text{ }}{i_0}\left[ {{c_R}*exp\left( {{\alpha _a}F\eta /RT} \right){i_0} - {c_O}*exp\left( { - {\alpha _c}F\eta /RT} \right)} \right]$$

where i_0_ stands for the exchange current density; c_R_ and c_O_ are the concentrations of the oxidized and reduced species (initial concentration of Na ion in the electrolyte is 1 M), respectively; upon deposition process, c_R_ maintains constant (1 M), while the value of c_O_ is given according to Nernst–Planck equation as described before [49]; value of the α_a_ and α_c_ is 0.5, respectively; and η, R and T are the overpotential, gas constant and temperature, respectively. Therefore, Na metal surface deposition behavior can be obtained based on Faraday’s law of electrolysis:

2 $${\text{V}}  =   - {\rm Mi/}\rho {\text{Fn }}$$

ρ and M are the density and molar mass of Na metal, respectively.

## Results and Discussion

### Microstructure and Morphology Characterizations

**Fig. 1:**
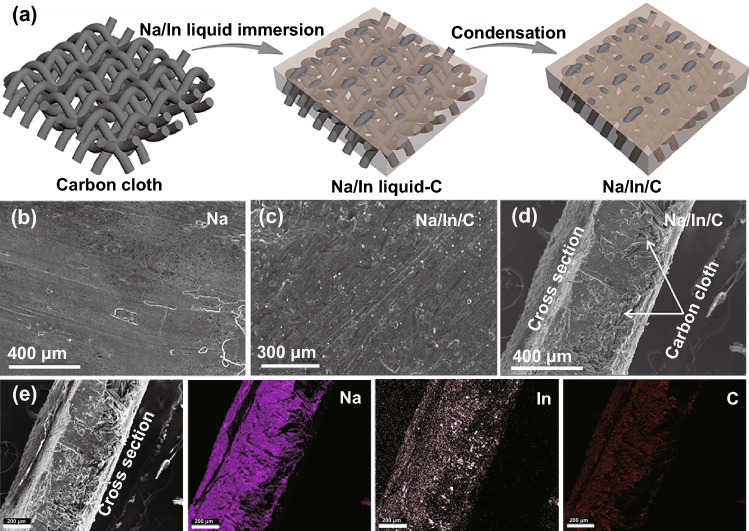
**a** Schematic image of the fabrication process of the Na/In/C composite. **b, c** SEM images of the pure Na metal the Na/In/C composite. **d** Cross-sectional SEM images of the Na/In/C composite. **e** Element mappings of the synthesized Na/In/C composite anode

As depicted in Fig. 1a, the Na/In/C composite was fabricated via a facile Na/In molten metal infusion method with a carbon cloth as the accommodation matrix and a subsequent condensation process (Na:In:C = 10:1:4 by weight). Driven by hybrid entropy and good wettability of the substrate, carbon cloth is uniformly covered by the molten Na/In metal, rendering a flat electrode surface similar to pure Na metal surface as presented in Fig. 1b-d. As shown in Fig. S2, XRD peaks confirm the existence of cubic NaIn and orthorhombic Na_2_In apart from the typical diffraction peaks of cubic Na metal. Furthermore, as shown in Fig. 1e, elemental mapping images confirm the uniform distributions of Na and In within the whole composite electrode.

### Electrochemical Testing of the Na Ion Deposition

**Fig. 2:**
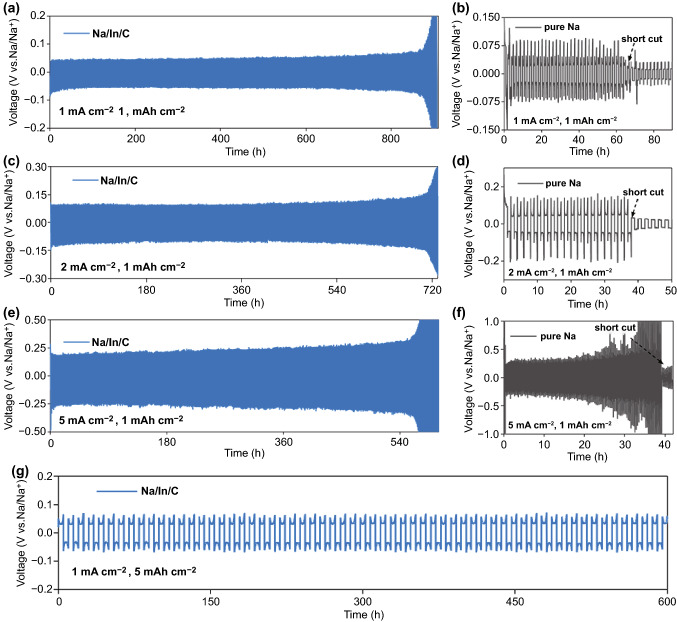
**a, c, e** Long-term Na ion plating/stripping profiles of the Na/In/C composite symmetrical cells at 1, 2 and 5 mA cm^−2^ with a capacity of 1 mAh cm^−2^. **b, d, f** Cycling performances of symmetrical cells assembled with pure Na metal at 1, 2 and 5 mA cm^−2^ with a capacity of 1 mAh cm^−2^. **g** Long-term Na ion deposition profiles of the Na/In/C composite symmetrical cell at 1 mA cm^−2^ with a high capacity of 5 mAh cm^−2^

**Fig. 3:**
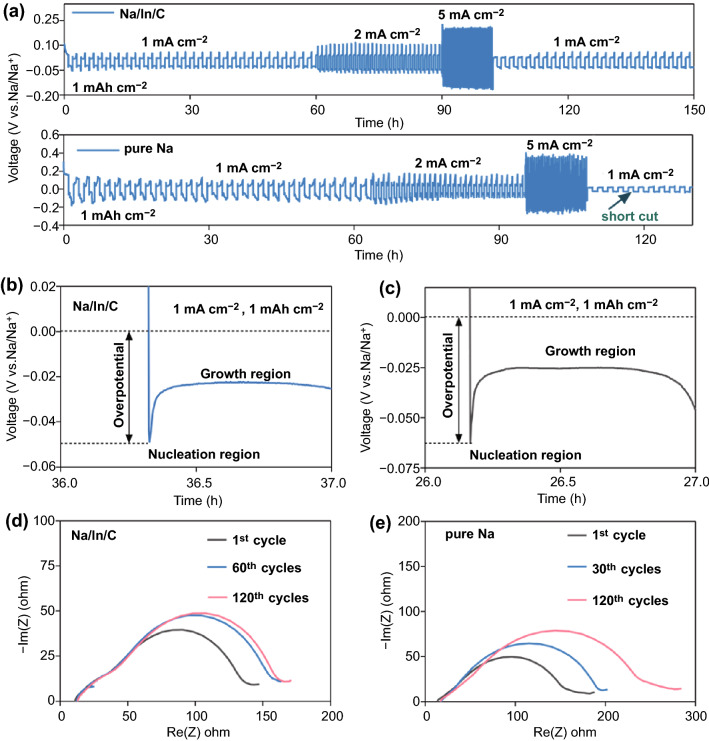
**a** Rate performances of the symmetric cells assembled with the Na/In/C composite and Na metal at 1, 2 and 5 mA cm^−2^. **b, c** Nucleation overpotentials for Na ion deposition in Na/In/C||Na/In/C and Na||Na cells at a current density of 1 mA cm^−2^, 1 mAh cm^−2^. **d, e** Electrochemical impedance analyses of the Na/In/C||Na/In/C cells and the Na||Na cells after 1, 60 and 120 cycles

Na ion plating/stripping properties of the Na/In/C and the pure Na metal were subsequently evaluated with symmetrical coin cells. 1.0 M NaClO_4_ in EC:PC (1:1 by volume) with 5 wt% FEC was employed as an electrolyte. As shown in Fig. 2a, c, cells cycled galvanostatically at 1 and 2 mA cm^−2^ (1 mAh cm^−2^) demonstrated stable deposition overvoltage of 51/100 mV and smooth voltage profiles over 870/710 h, respectively. Intriguingly, even under a harsh condition of 5 mA cm^−2^ (Fig. 2e), the cell still has a long-term cycling life over 560 h. Of note, upon extensive cycling process at 5 mA cm^−2^, a thick SEI with relative low quality (compared with 1 and 2 mA cm^−2^) and gradually increasing thickness will cover the whole anode surface [50], hence resulting in the formation of protrusions and islands (Fig. S3b, c) as well as slow Na ion transport kinetics within the SEI (Fig. S3a). As a result, larger overpotential would be needed to maintain a constant applied deposition current based on the Butler–Volmer equation, thereby leading to a progressively increasing overvoltage as presented in Fig. 2e [51]. It is also remarkable that cells with the Na/In/C electrode can also deliver a long life span over 300 h at 1 and 2 mA cm^−2^ with a higher capacity of 3 mAh cm^−2^ as shown in Fig. S4a, b. Moreover, even at a harsh testing condition of 5 mAh cm^−2^, the Na/In/C electrode can still retain an impressive cycling stability over 600/300 h at 1 and 5 mA cm^−2^ (Figs. 2g and S4c). Notably, the Na/In/C electrode demonstrates better stabilities among recently reported Na metal batteries cycled in the common EC/PC electrolyte (Fig. S5). By comparison, as illustrated in Fig. 2b, d, f, it can be seen that cells assembled with pure Na metal electrodes experience much larger voltage hysteresis of 82, 156 and 340 mV at 1, 2 and 5 mA cm^−2^ (1 mAh cm^−2^). Even worse, these cells all display an abrupt voltage drop (Fig. 2b, d, f,) in a few cycling hours, indicative of the soft short circuit originated from penetration by notorious sodium dendrites. Moreover, rate capability of the symmetrical Na/In/C and Na cells are depicted in Fig. 3a. Apparently, as the current density changing from 1 to 5 mA cm^−2^, the cell utilizing Na/In/C composite electrodes show low voltage fluctuation and highly stable Na ion deposition. In parallel, long-term cycling stabilities of cells employing Na/In/C anodes with different Na:In weight ratios (8:1 and 12:1) were also evaluated. As shown in Fig. S6a, d, both Na/In/C samples (8:1 and 12:1) demonstrate the distinct presence of natrophilic NaIn and Na_2_In phases. As to the Na/In/C anode with a ratio of 12:1, it is obvious that cells show large voltage fluctuations and unstable charge/charge profiles at 1 and 5 mA cm^−2^ (1 mAh cm^−2^) (Fig. S6b, c). This could be mainly ascribed to the reduced natrophilic NaIn and Na_2_In phases compared with Na/In/C anode with a ratio of 10:1. Albeit cells utilizing Na/In/C anode (8:1) can deliver similar cycling stability at 1 and 5 mA cm^−2^ (1 mAh cm^−2^) (Fig. S6e, f) as those employing Na/In/C anode (10:1), fabricating Na/In/C anode with a ratio of 10:1 is much more preferable on account of higher prices of In.

As expected, cell with pure Na metal exhibits a poor tolerance to the rate testing condition and a sudden voltage drop associated with short circuit occurs (Fig. 3a), meaning that Na metal undergoes a much more severe corrosion in the EC/PC electrolyte. Under the 2 and 5 mA cm^−2^ testing (1 mAh cm^−2^) conditions, it can be witnessed that cells with the Na/In/C composite electrodes demonstrate a much smaller nucleation overpotential entailed for Na ion plating comparing with the cells with pure Na metal electrodes (Fig. S7a-d). Interestingly, it is found that the pure Na metal and the Na/In/C anode possess similar growth overpotentials (Na: ~ 25 mV, Na/In/C: ~ 20 mV) at 1 mA cm^−2^ in the initial tens of cycles (Fig. 3b, c), which may originate from their similar SEI thicknesses and Na ion diffusion kinetics within SEI under a low Na ion deposition current [50]. However, upon long-term cycling process, reactive Na metal demonstrates unstable Na ion deposition process and even short cut, which can be largely attributed to the poor SEI quality and dendrites formation as shown schematically in Fig. S8. This suggests that the Na–In alloy can significantly decrease Na ion nucleation barrier and promote Na ion nucleation and propagation kinetics [10].

Electrochemical impedance of the symmetric cells with Na/In/C and Na metal electrodes was further measured with EIS. Figure 3d, e shows Nyquist plots of the Na/In/C||Na/In/C cells and Na||Na cells after 1, 60 and 120 cycles at 1 mA cm^−2^ (1 mAh cm^−2^). From 60 to 120 cycles, EIS profiles for Na/In/C||Na/In/C cells show little changes, while those of the Na||Na cells change significantly. *R*_SEI_ and *R*_ct_, representing SEI quality and charge transfer dynamics, were obtained by fitting the corresponding EIS profiles as manifested by Fig. S9a, b. Clearly, cell with the Na/In/C composite electrode presents low *R*_SEI_ of 21.75 Ω and *R*_ct_ of 71.44 Ω (Fig. S9a) after initial cycling. After 60 and 120 cycles, these *R*_SEI_ and *R*_ct_ values undergoes only mild increases upon cycling. In contrast, although the cell with Na metal electrode demonstrates a similar *R*_SEI_ (23.86 Ω) and *R*_ct_ of 72.89 Ω (Fig. S9a) after first cycle, conspicuous increases in *R*_SEI_ and *R*_ct_ values are shown in Fig. S9b after 60 and 120 cycles, indicating that the Na/In/C electrodes possess much improved charge transfer dynamics and better chemical stability in the common EC/PC electrolyte.

### Morphology Variation and Surface Analyses

**Fig. 4:**
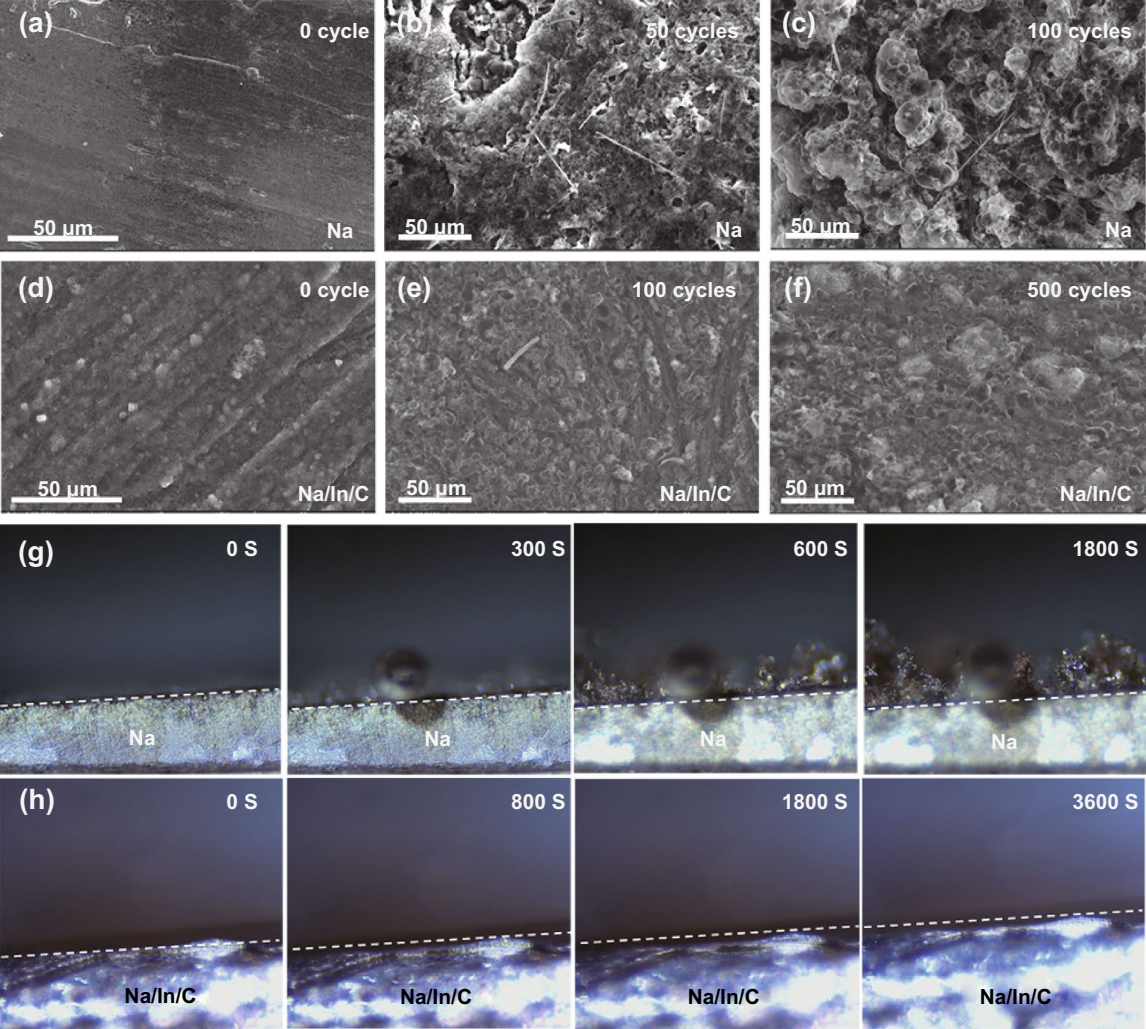
**a–c** SEM images of the cycled Na metal anode at 1 mA cm^−2^ with a capacity of 1 mAh cm^−2^ after 0, 50 and 100 cycles. **d-f** SEM images of the cycled Na/In/C composite electrode at 1 mA cm^−2^ with a capacity of 1 mAh cm^−2^ after 0, 100 and 500 cycles. **g, h** In situ optical microscopic images of Na ion deposition process on pure Na metal and the Na/In/C composite electrode at 1 mA cm^−2^, 1 mAh cm^−2^

Morphological evolutions of the Na metal and the Na/In/C composite electrode after various cycles (1 mA cm^−2^, 1 mAh cm^−2^) were investigated using SEM and in situ optical microscopy. As shown in Fig. 4a-c, dendritic and porous Na deposition emerges after 50 and 100 cycles, which can be further revealed by in situ optical microscopy visualizing the deposited Na surface (Fig. 4g). On the contrary, the Na/In/C electrode preserves a dendrite-free and unscathed morphology after 100 and 500 cycles (Fig. 4d-f). Upon cycling at higher current densities of 2 and 5 mA cm^−2^ (Fig. S10a-f), the Na/In/C composite electrode maintains similar morphologies as that cycled at 1 mA cm^−2^ (Fig. 4d-f), while pure Na metal anode suffers from alike dendritic and porous Na deposition after 100 and 500 cycles (Fig. S11a-f). Besides, in situ optical microscopy (Figs. 4h and S10g, h) shows that the Na/In/C surface is smooth without forming any detectable sodium protuberance and dendrite, again confirming remarkable interfacial stability of the Na/In/C electrode in the EC/PC electrolyte.

**Fig. 5:**
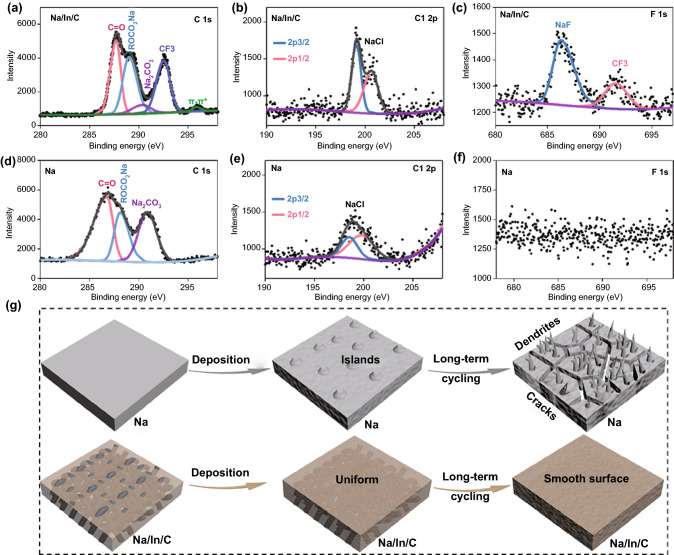
High-resolution XPS profiles: **a, d** C 1 s, **b, e** Cl 2p and **c, f** F 1 s of the Na metal and the Na/In/C composite electrodes after 50 cycles at 1 mA cm^−2^, 1 mAh cm^−2^. **g** Schematic of Na ion deposition behavior on a pure Na metal anode and a Na/In/C composite anode. Na ion tends to selectively deposit on defects and protruded area leading to the formation of cracks and dendrites for pure Na anode, while Na ions prefer to smoothly and uniformly deposit for Na/In/C composite anode

Other than the post-cycling morphology evolution analysis, interfacial chemistry of the Na and the Na/In/C composite electrodes after 50 cycles at 1 mA cm^−2^ (1 mAh cm^−2^) was studied with XPS (Figs. 5 and S12). As shown in the C 1 s spectra of the pure Na metal (Fig. 5d), several clear peaks located at 286.7, 288.9 and 290.4 eV can be assigned to -C-O-, ROCO_2_Na and Na_2_CO_3_ [39]. Interestingly, except the existence of the above products, it was worth mentioning that one extra peak at 292.5 eV related to –CF_3_ can be observed in the C 1 s spectra of the Na/In/C electrode (Fig. 5a). Apart from this difference, C 1 s signals associated with the decomposed carbonate electrolyte are stronger for the pure Na electrode. Additionally, compared with pure Na metal (Fig. 5e, f), the Na/In/C electrode demonstrates an intensive signal of the F 1 s (Fig. 5c) and Cl 2p spectra (Fig. 5b), suggesting apparent enrichments of NaF and NaCl products in the SEI component [51]. The absence of F signal of the pure Na metal anode after 50 cycles can be largely attributed to the continuous breaking of SEI on the Na dendrite structures (Figs. 4a-c and S11a-f, S13), leading to the unnecessary consumption of electrolyte and thus causing ultralow contents of preferable NaF and –CF_3_ [51]. Furthermore, it is apparent that peaks located at 441.3/441.6 and 449.2/449.8 eV (Fig. S14) can be ascribed to the presence of natrophilic NaIn and Na_2_In phases on the Na/In/C composite electrode interface [52], which are conducive to promoting Na ion deposition stability as verified by both experimental analyses and DFT simulations. It is thus reasonable to consider that the Na/In/C electrode possesses a much more robust SEI structure and interface stability in the EC/PC electrolyte. According to the above analyses, it is reasonably accepted that Na ions preferentially deposit at the protuberances upon cycling (Fig. 5g), which ultimately result in the formation of cracks and dendrites. By contrast, the presence of In can improve Na ion nucleation kinetics and guide uniform Na ion deposition (Fig. 5g), thus effectively suppress Na dendrites growth and restrain the volume bulge of Na metal during long-term cycling.

### Sodium Ion Deposition Reversibility Analysis

**Fig. 6:**
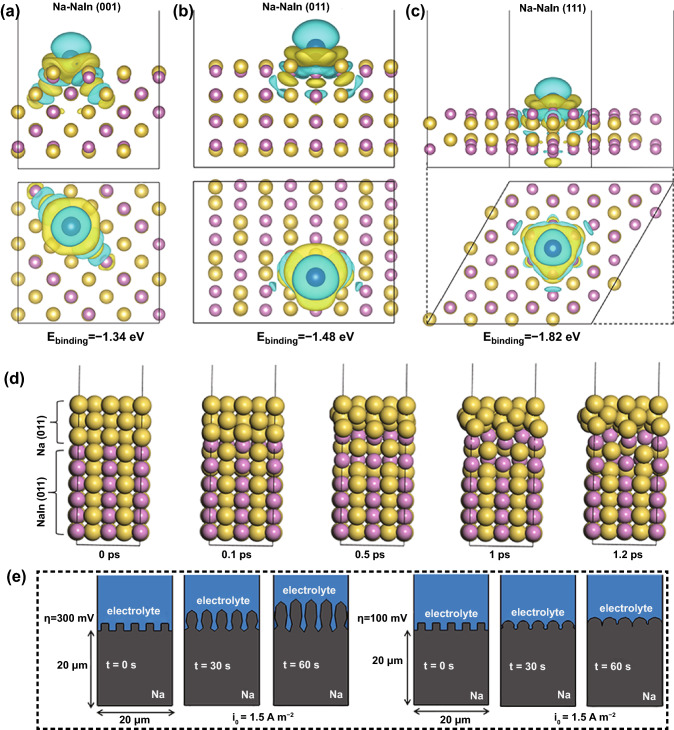
**a–c** Charge differential density and the corresponding binding energies of the adsorbed Na atom on (100), (110) and (111) crystal planes of NaIn. **d** AIMD calculation results of Na ion plating kinetics on the (110) surface of NaIn at 0.1, 0.5, 1 and 2 ps. **e** Finite element simulations of Na metal electrode morphology evolution features with square-shaped bumps at an exchange density of 1.5 A m^−2^ with various overpotential and time (0 s, 30 s, 60 s)

To obtain a better understanding of the superior sodium ion deposition reversibility on the Na/In/C electrode surface, DFT, AIMD and FE simulations were carried out to delineate the Na ion binding sites/energy, plating kinetics and deposition morphology evolution process. Initial adsorption structure models of Na–Na_2_In and Na–NaIn are provided in Fig. S15. As summarized in Fig. 6a-c, optimized binding energies between a Na ad-atom (blue color) and NaIn surfaces are -1.34 (100), -1.48 (110) and -1.82 eV (111), whereas these values on pure Na surfaces are -1.089 (100), -1.067 (110) and -0.761 eV (111), respectively (Fig. S16). These show that NaIn owns an intrinsic natrophilic merit and this can largely reduce Na nucleation energy and homogenize the Na^+^ deposition flux. Meanwhile, the (311) crystal plane of Na_2_In also shows preferable natrophilic feature as shown by its high binding energy of -1.93 eV (Fig. S17). A lattice matching (100) interface between Na and NaIn is built to further ascertain Na ion deposition stability via AIMD simulation (Fig. 6d). As evidenced, a smooth and compact interface is sustained during the whole AIMD simulation process, thus significantly facilitating Na ion plating/stripping kinetics and removing morphological instability. In sharp contrast, porous structures are formed on the pure Na metal surface as shown in Fig. S18, which agrees well with the experimental results. Finite element simulations were further used to study evolution processes of the electrode surface at various overpotentials and time (0, 30 and 60 s). Initially, Na metal surface with square-shaped bumps was intentionally fabricated to examine Na ion deposition behavior under different overpotential (Fig. 6e). Noticeably, larger overpotential (300 meV) induces conspicuous preferential deposition, leading to severe directional growth, morphology irregularity and eventually a porous structure. However, at a lower overpotential (100 meV), unwanted porous and dendritic growth of Na metal is effectively suppressed. In addition to the effect of overpotential on Na ion deposition kinetics, initial bump shapes also play a decisive role on the Na ion plating behavior. For instance, grave dendritic growth and preferential Na ion plating occurred for the triangle-shaped bumps even at a low overpotential (100 meV), which can be ascribed to the concentrated equipotential lines at the sharp corners (Fig. S19). Adversely, the presence of regular circle-shaped bumps on the Na metal surface brought about smooth surface evolution at high 300 meV overpotential (Fig. S20). Additionally, higher exchange current density (*i*_0_) (based on the Tafel profiles analyses shown in Fig. S21a, b, *i*_0_ values of the pure Na metal and the Na/In/C anode are 0.158 and 0.332 mA cm^−2^, respectively) promotes smooth Na ion deposition even at larger overpotential as shown in Fig. S22 for Na/In. All these theoretical analyses evidently reveal the reversible Na ion deposition mechanism on the Na/In/C electrode (lower overpotential and larger exchange current density values) surface, matching well with prior electrochemical and chemical results.

### Feasibility of the Na/In/C Composite Electrode in Sodium Metal Full Cell

**Fig. 7:**
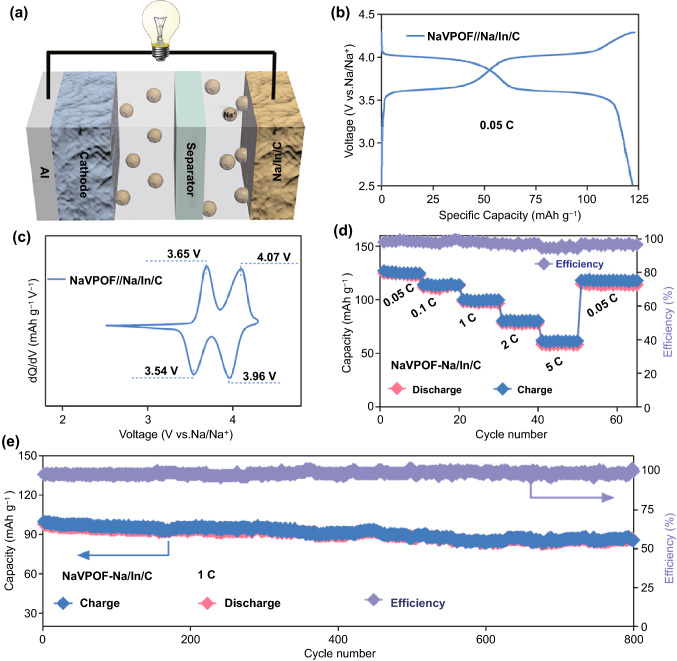
**a** Schematic illustration of a NaVPOF||Na/In/C sodium metal full battery. Electrochemical performances of the metal full battery: **b** voltage profile; **c** CV curve at a scan rate of 0.5 mV s-1; **d** rate performance at 2.5–4.3 V; **e** long-term cycling capability at 1 C within 2.5–4.3 V

To further elaborate the suitability of the Na/In/C composite electrode in EC/PC electrolyte (1 M NaClO_4_ in EC/PC), Na/In/C composite and Na foil were, respectively, paired with a high-voltage Na_3_V_2_O_2_(PO_4_)_2_F (NaVPOF) cathode to construct full coin cells. According to the area mass loading of the Na, Na/In/C and NaVPOF (experimental section) and the theoretical capacity of Na (1166 mAh g^−1^)/NaVPOF (130.7 mAh g^−1^, 2 ~ 4.5 V), ratios of the negative capacity to the positive capacity (N/P ratio) for the Na||NaVPOF and Na/In/C||NaVPOF full batteries are 6.2 and 4.1, respectively. XRD and SEM images of the NaVPOF cathode synthesized via a facile hydrothermal method are shown in Fig. S23a, b. Figure 7a presents specific illustration of sodium metal full batteries paired a high-voltage NaVPOF cathode with the Na/In/C composite anode. As exhibited in Fig. 7b, c, the cell demonstrates a smooth voltage profile at 0.05 C and sharp redox peaks with a scan rate of 0.5 mV s^−1^ ranging from 2.5 to 4.3 V (vs Na/Na^+^). Moreover, the NaVPOF-Na/In/C cell yields highly reversible charge capacities of 124, 115, 101, 80 and 61 mAh g^−1^ at rates of 0.05, 0.1, 1, 2 and 5 C, respectively (Fig. 7d). More promisingly, the cell is observed to be able to deliver a specific capacity of 88.4 mAh g^−1^ after 800 cycles with a capacity retention of 87.6% at 1 C rate, which contributes to a low capacity attenuation of 0.019% per cycle (Fig. 7e). Unsurprisingly, the NaVPOF-Na cell shows unsteady charge/discharge curve (Fig. S24a), large voltage polarization (Fig. S24b) and inferior cycling capability at 1 C (Fig. S24c). These results reinforce the much better performance of the Na/In/C composite electrode in sodium metal full batteries in the common EC/PC electrolyte.

## Conclusions

In summary, a Na/In/C composite anode was synthesized by infusing metallic Na and In on a carbon cloth scaffold. As a result, symmetric cells with Na/In/C electrode manifests low-voltage hysteresis and impressive long-term cycling stability at 1 mA cm^−2^ (> 870 h), 2 mA cm^−2^ (> 710 h) and 5 mA cm^−2^ (> 560 h), respectively, with a capacity of 1 mAh cm^−2^ in a typical EC/PC electrolyte. SEM and in situ optical microscopy analyses clearly unravel that a dendrite-free and smooth morphology can be obtained in the Na/In/C composite symmetric cells. Furthermore, DFT, AIMD and FE calculations uncover the intrinsic mechanism for the reversible Na ion deposition behavior on the Na/In/C electrode. Moreover, a full sodium metal cell with a NaVPOF cathode and a Na/In/C anode presents enhanced Na ion deposition kinetics and electrochemical cycling capability. The present work offers a novel alloy strategy into the modification of Na metal chemistry for improving Na ion plating/stripping stability in the common EC/PC electrolyte.

## Supplementary Information

Below is the link to the electronic supplementary material.

Supplementary file1 (PDF 2754 kb)
