# Supplementary material for: Fabricating Na/In/C Composite Anode with Natrophilic Na–In Alloy Enables Superior Na Ion Deposition in the EC/PC Electrolyte
Source: Nanomicro Lett. 2021 Dec 9;14:23. doi: 10.1007/s40820-021-00756-7 (PMC8660945; doi:10.1007/s40820-021-00756-7)
Supplement: Supplementary file 1 — Supplementary file1 (PDF 2754 kb) [file 40820_2021_756_MOESM1_ESM.pdf]

Supporting Information for

Fabricating Na/In/C Composite Anode with Natrophilic Na-In Alloy Enables Superior Na Ion Deposition in the EC/PC Electrolyte

Hui Wang[1, 2], Yan Wu[2], Ye Wang[1], Tingting Xu[1], Dezhi Kong[1], Yang Jiang[3], Di Wu[1], Yongbing Tang[4], Xinjian Li[1, *], Chun-Sing Lee[2, *]

[1]Key Laboratory of Material Physics of Ministry of Education, School of Physics and Microelectronics, Zhengzhou University, Zhengzhou 450052, P. R. China

[2]Center of Super-Diamond and Advanced Films (COSDAF) and Department of Chemistry, City University of Hong Kong, Hong Kong SAR 999077, P. R. China

[3]School of Materials Science and Engineering, Hefei University of Technology, Hefei, Anhui 230009, P. R. China

[4]Shenzhen Institutes of Advanced Technology, Chinese Academy of Sciences, Shenzhen 518055, P. R. China

*Corresponding authors. E-mail: lixj@zzu.edu.cn (Xinjian Li); apcslee@cityu.edu.hk (Chun-Sing Lee)

Supplementary Figures

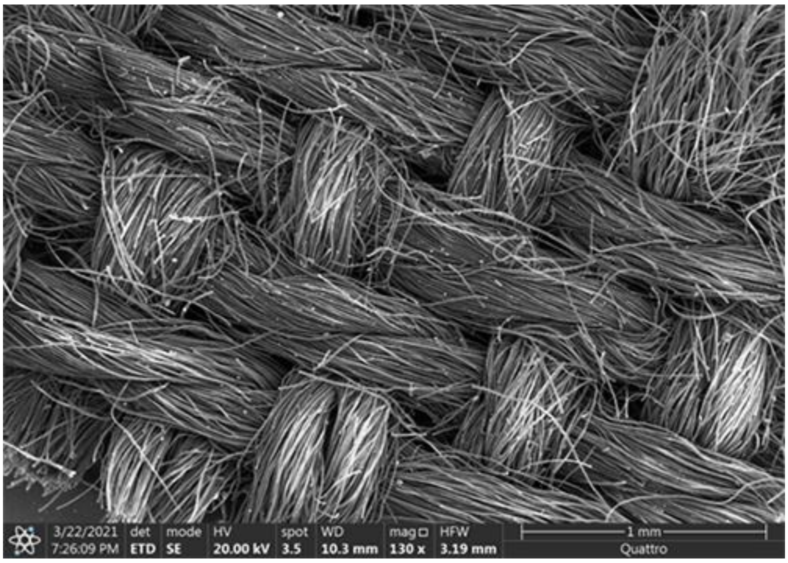

Fig. S1 SEM image of the carbon cloth

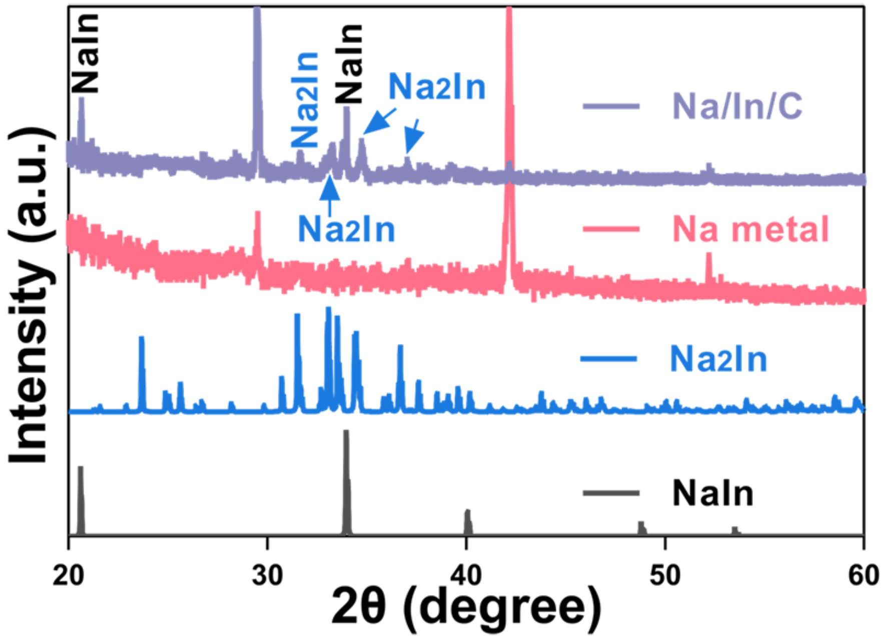

Fig. S2 XRD patterns of the pure Na metal and the Na/In/C composite

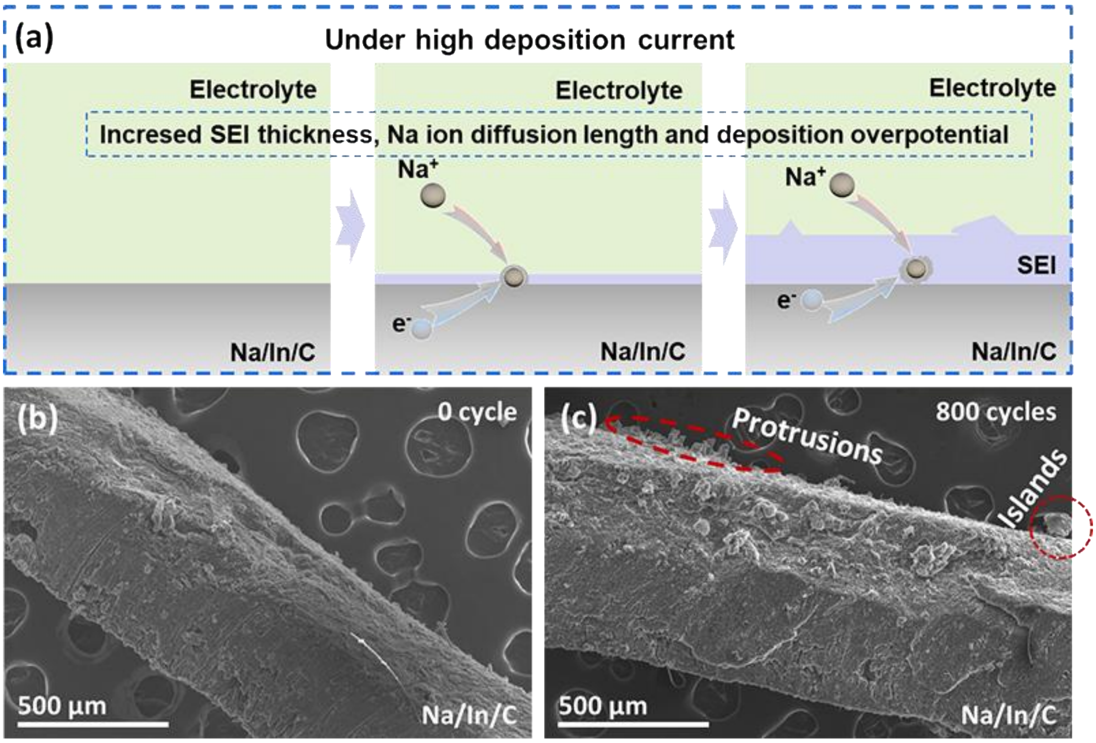

Fig. S3 (**a**) A schematic image showing Na ion deposition on the interface between Na metal and SEI under high deposition currents. (**b**) and (**c**) Cross-sectional SEM images of the Na/In/C composite after 0 and 800 cycles at 5 mA cm^{-2} with a capacity of 1 mAh cm^{-2}

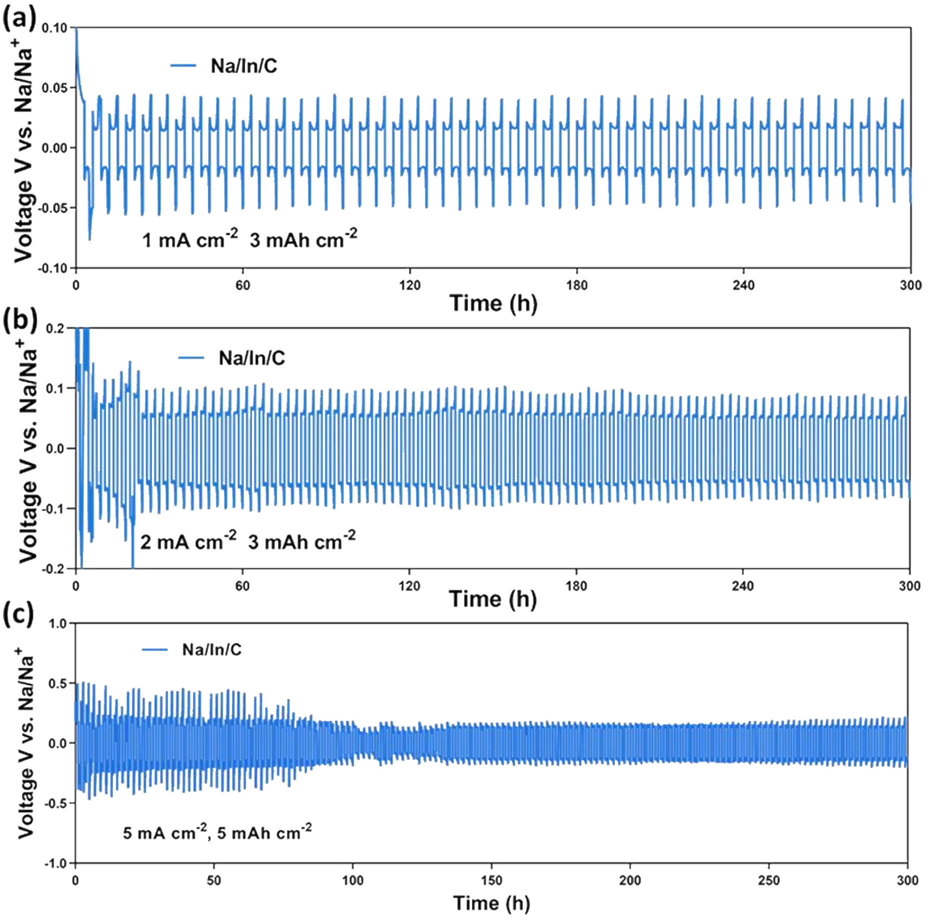

Fig. S4 (**a**) and (**b**) Long-term Na ion plating/stripping profiles of the Na/In/C composite symmetrical cells at 1 and 2 mA cm^{-2} with a high capacity of 3 mAh cm^{-2} respectively. (**c**) Long-term Na ion plating/stripping profiles of the Na/In/C composite symmetrical cell at 5 mA cm^{-2} with a high capacity of 5 mAh cm^{-2}

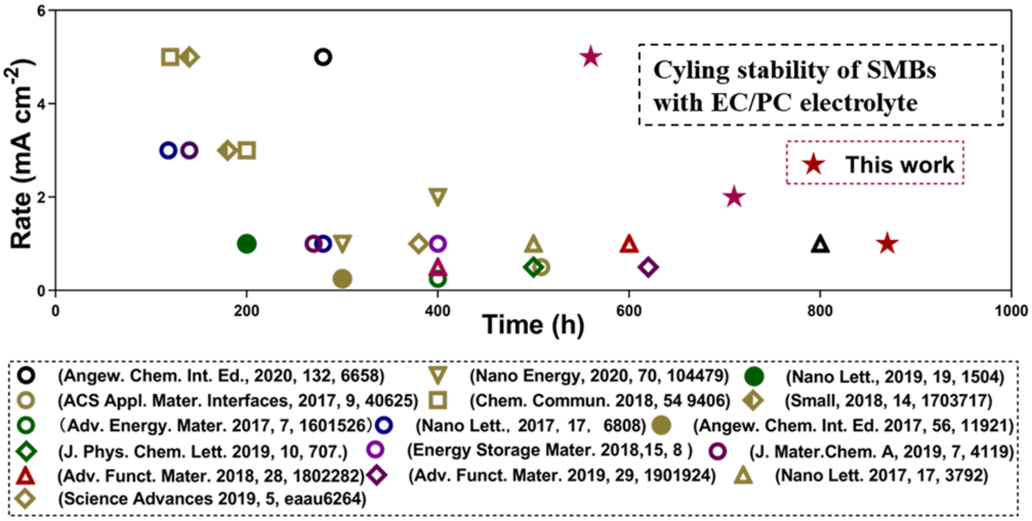

Fig. S5 Stability comparison of recently reported anodes upon the representative papers concerning Na ion deposition stability with a capacity of 1 mAh cm^{-2} in EC/PC electrolytes.

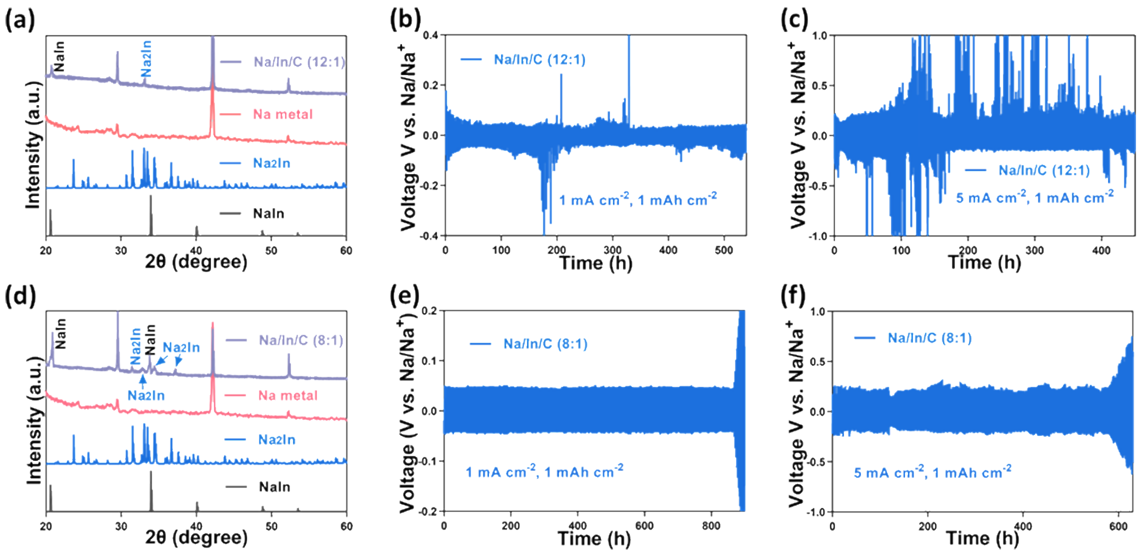

Fig. S6 (**a**) and (**d**) XRD patterns of the Na/In/C composite with Na:In weight ratis of 12:1 and 8:1. (**b**), (**c**), (**e**) and (**f**) Long-term Na ion plating/stripping profiles of the corresponding Na/In/C composite symmetrical cells at 1 and 5 mA cm^{-2} with a capacity of 1 mAh cm^{-2}

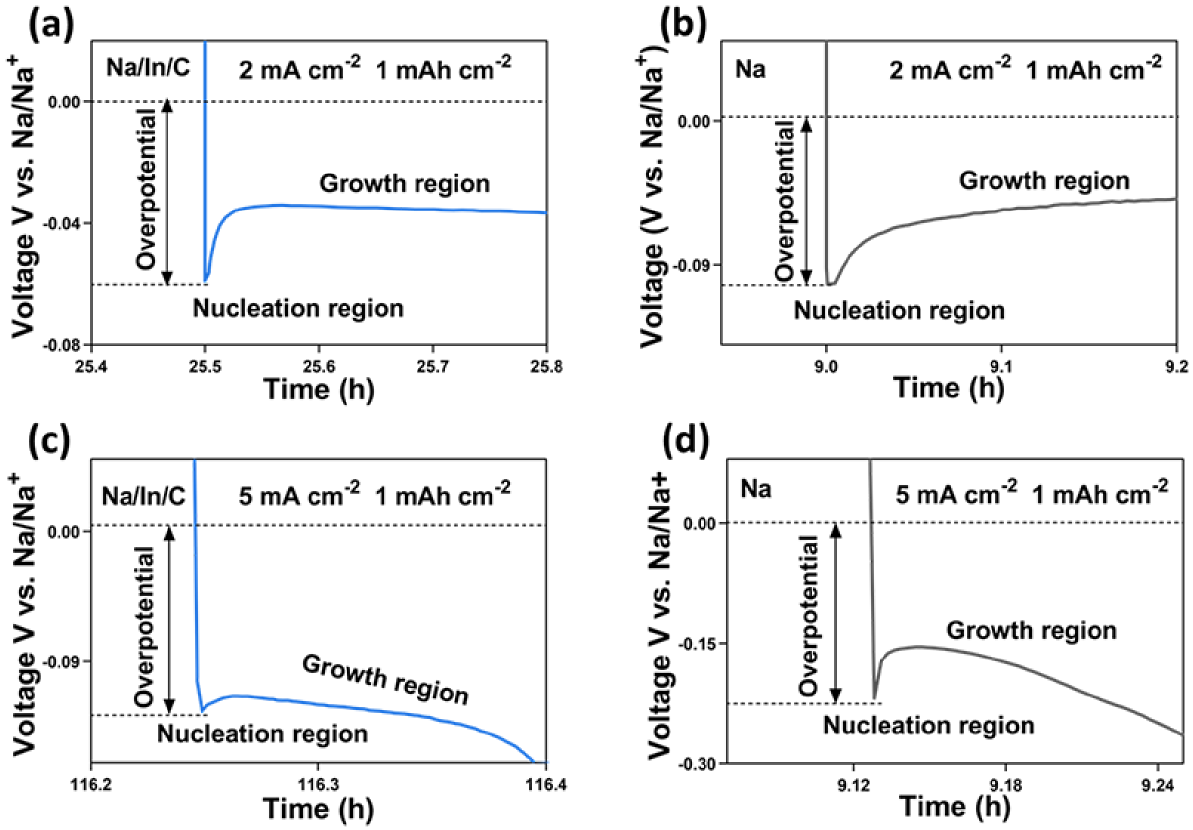

Fig. S7 (**a-d**) Nucleation overpotentials associated with Na ion deposition in Na/In/C||Na/In/C and Na||Na cells at a current density of 2/5 mA cm^{-2}, 1 mAh cm^{-2}

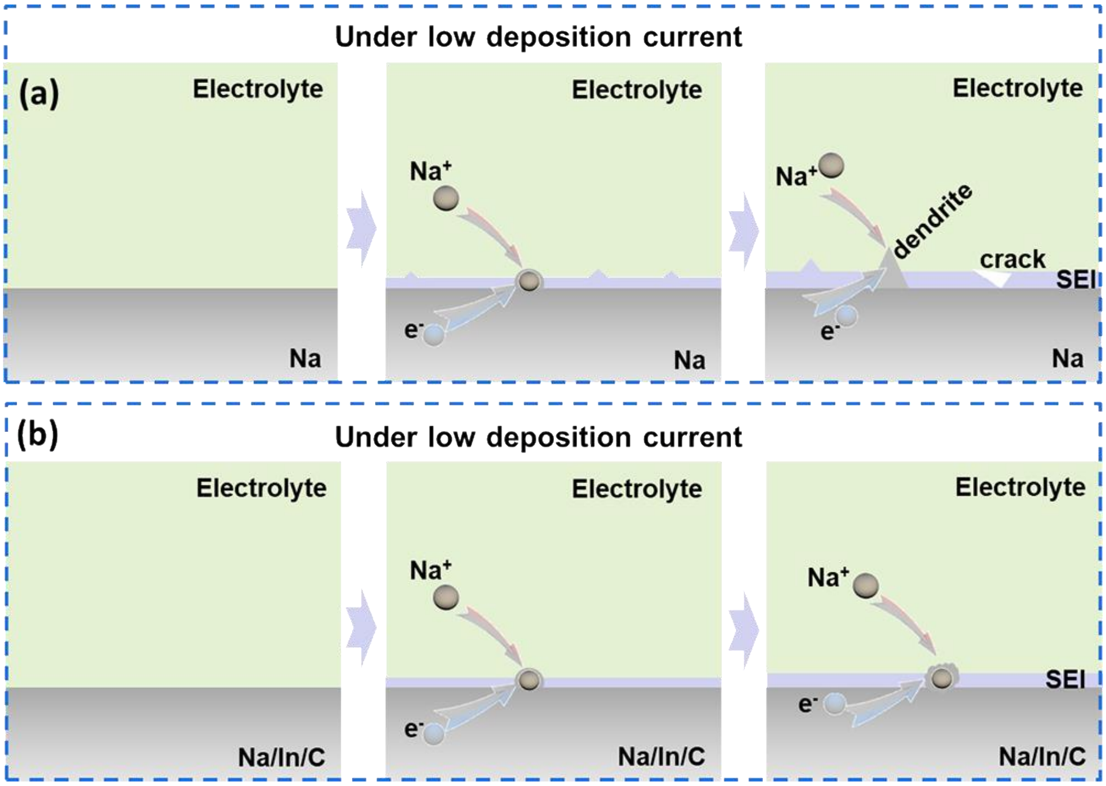

Fig. S8 (**a**) and (**b**) Schematic diagrams of the Na ion deposition on the interface between Na metal and SEI, Na/In/C and SEI under low deposition current

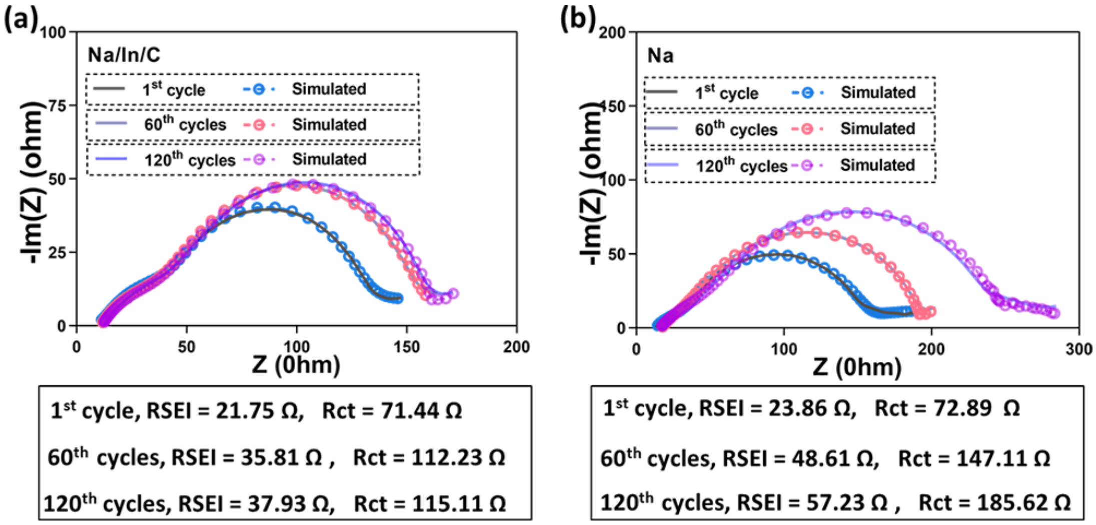

Fig. S9 EIS profiles and the corresponding resistance values obtained by modeling the experimental impedance of the symmetric Na/In/C||Na/In/C cells and Na||Na cells after 1, 60 and 120 cycles at 1 mA cm^{-2}, 1 mAh cm^{-2}

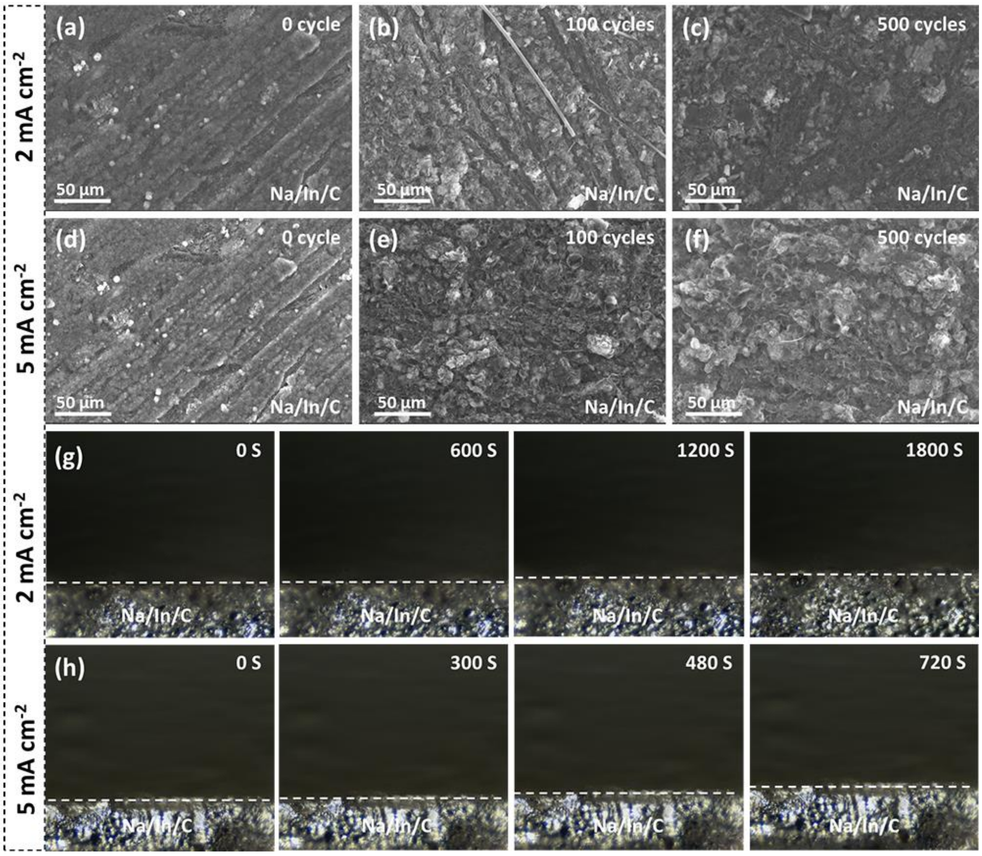

Fig. S10 (**a-c**) give SEM images of the cycled Na/In/C electrode at 2 mA cm^{-2} with a capacity of 1 mAh cm^{-2} after 0, 100 and 500 cycles. (**d-f**) demonstrate SEM images of the cycled Na/In/C electrode at 5 mA cm^{-2} with a capacity of 1 mAh cm^{-2} after 0, 100 and 500 cycles. (**g, h**) present in-situ optical microscopy images of Na ion deposition process upon the Na/In/C composite electrodes surface respectively at 2 and 5 mA cm^{-2} (1 mAh cm^{-2})

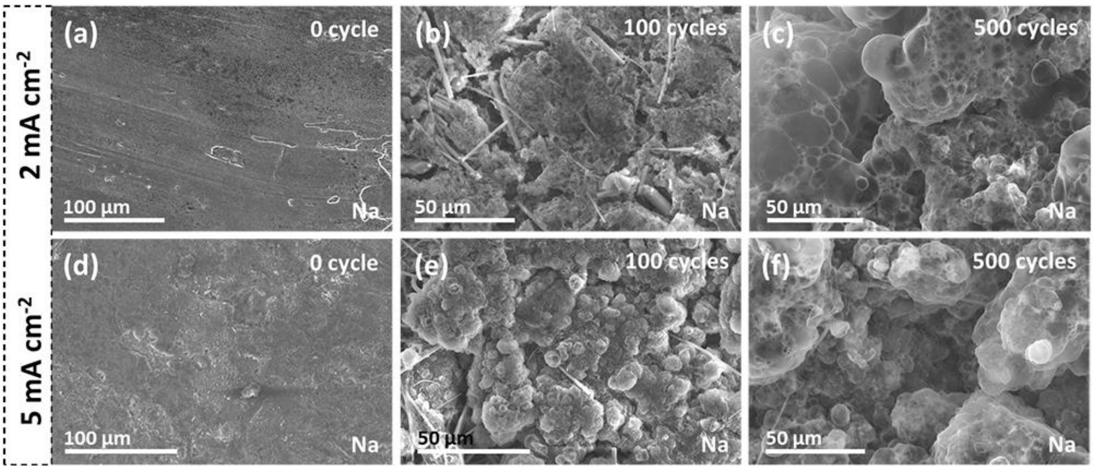

Fig. S11 (**a-c**) SEM images of the cycled pure Na electrode at 2 mA cm^{-2} with a capacity of 1 mAh cm^{-2} after 0, 100 and 500 cycles. (**d-f**) SEM images of the cycled pure Na electrode at 5 mA cm^{-2} with a capacity of 1 mAh cm^{-2} after 0, 100 and 500 cycles

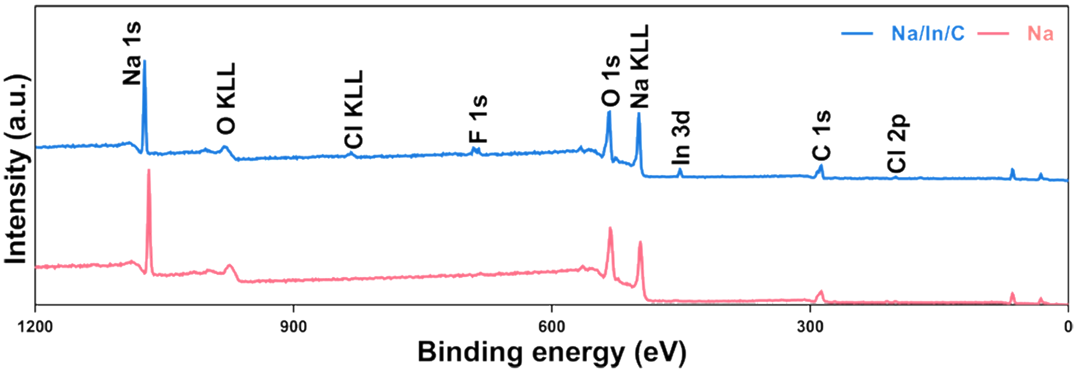

Fig. S12 XPS survey results of the Na metal and the Na/In/C composite electrodes after 50 cycles at 1 mA cm^{-2}, 1 mAh cm^{-2}

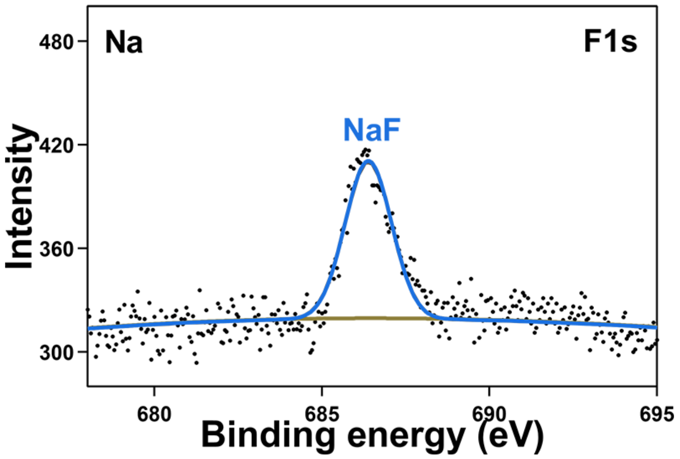

Fig. S13 High-resolution XPS profile of the F1s for the pure Na metal anode after 2 cycles at 1 mA cm^{-2}, 1 mAh cm^{-2}

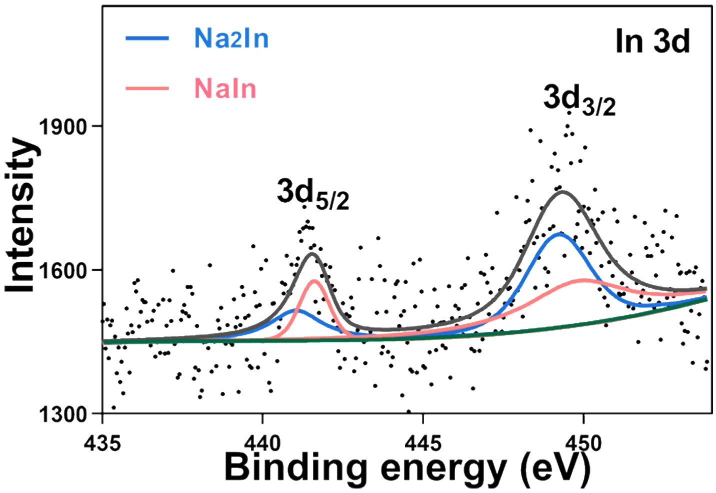

Fig. S14 High-resolution XPS profile of the In3d for the Na/In/C composite electrode after 50 cycles at 1 mA cm^{-2}, 1 mAh cm^{-2}

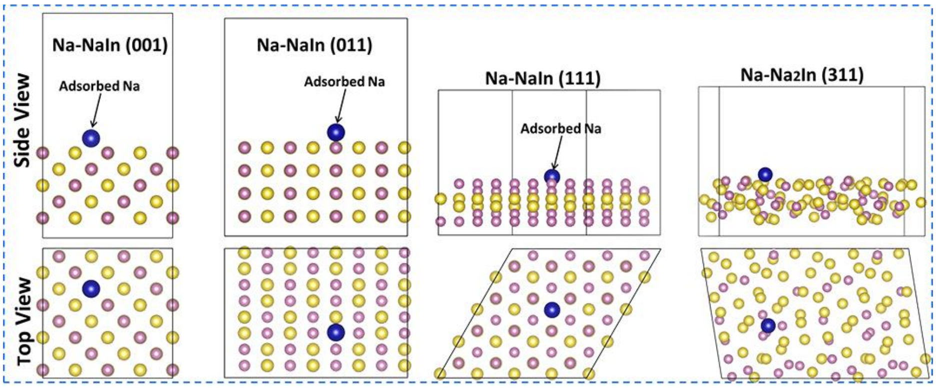

Fig. S15 Initial adsorption structure models of Na-Na_2In and Na-NaIn

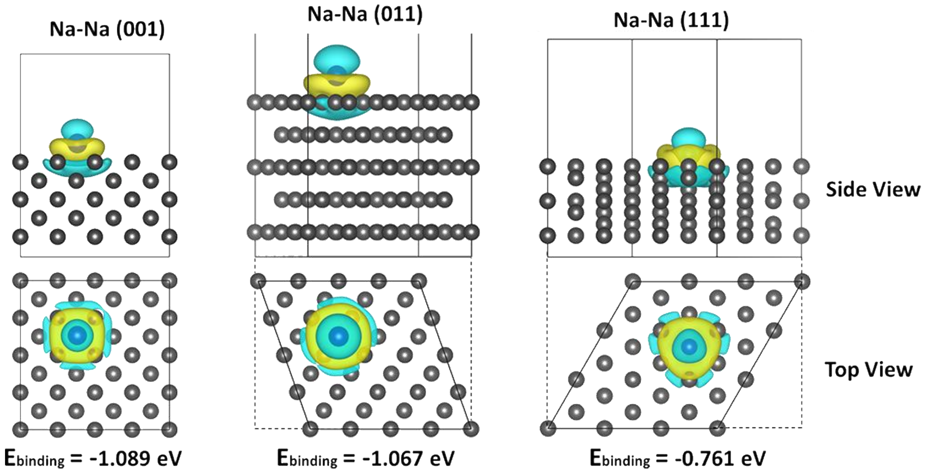

Fig. S16 Charge differential density and the corresponding binding energies of the adsorbed Na atom with the (100), (110) and (111) crystal planes of pure Na metal

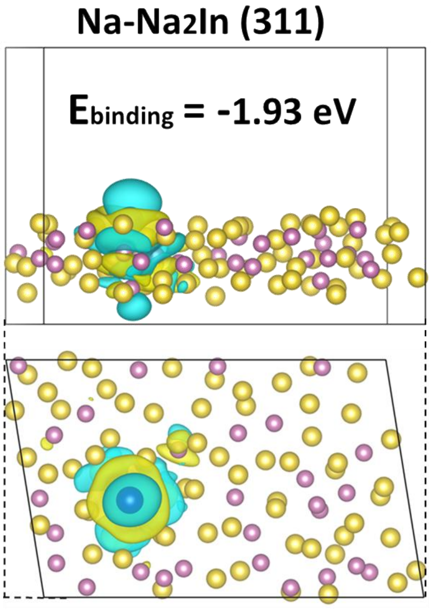

Fig. S17 Charge differential density and the corresponding binding energies of the adsorbed Na atom with the (311) crystal plane of Na_2In

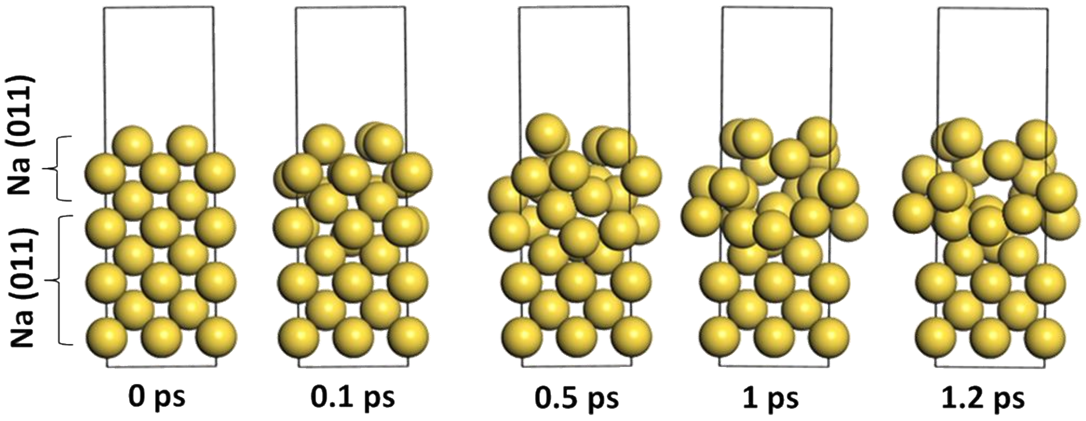

Fig. S18 AIMD calculation results of the Na ion plating kinetics on the (110) surface of Na metal at 0.1, 0.5, 1 and 2 ps

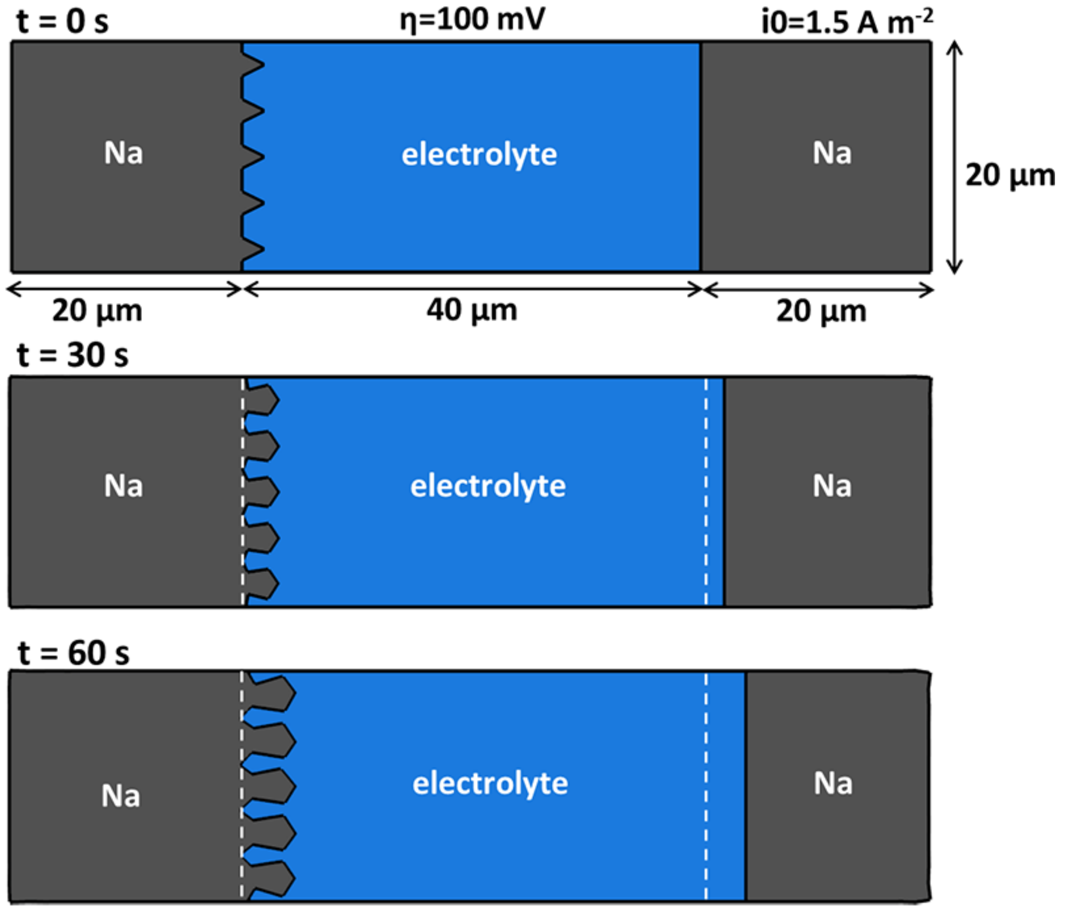

Fig. S19 Finite element simulations of Na metal electrode morphology evolution features with triangular-shaped bumps at various time (0 s, 30 s, 60 s) with a 100 meV overpotential and an exchange density of 1.5 A m^{-2}

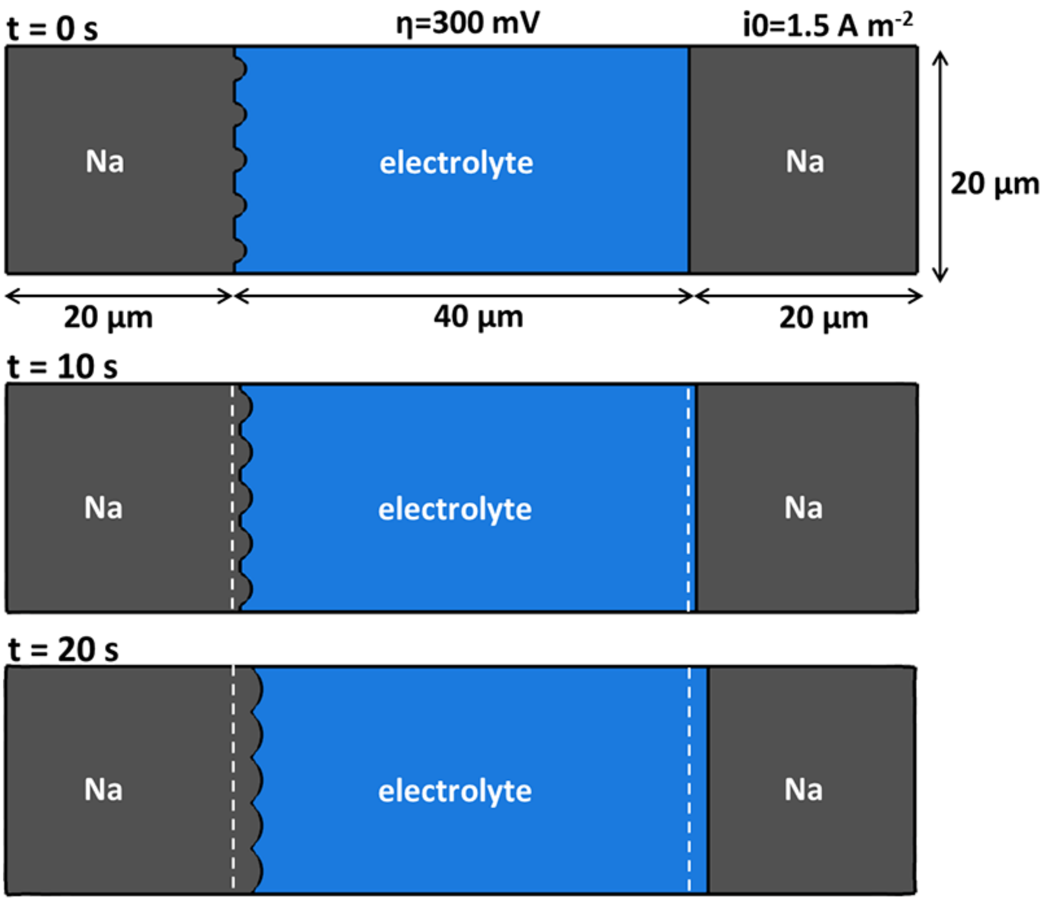

Fig. S20 Finite element simulations of Na metal electrode morphology evolution features with circular-shaped bumps at various time (0 s, 30 s, 60 s) with a 300 meV overpotential an exchange density of 1.5 A m^{-2}

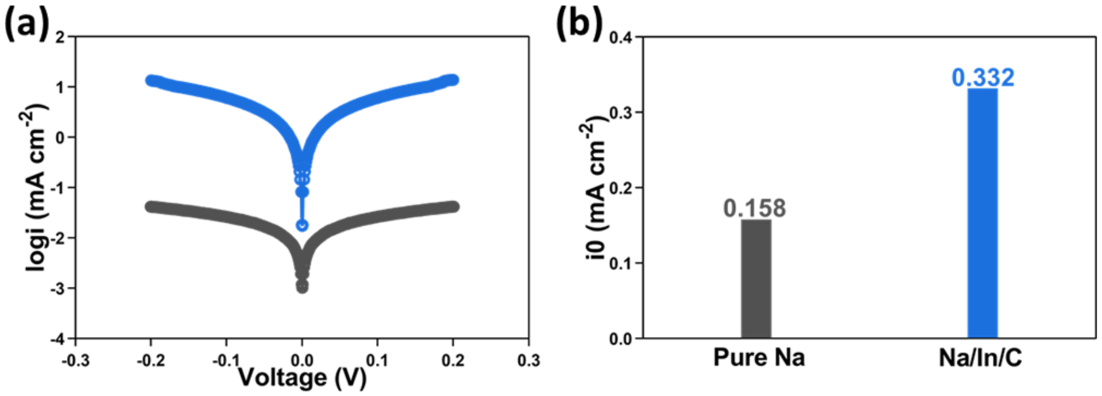

Fig. S21 (**a, b**) Tafel profiles and the corresponding i_0 values of the pure Na metal and the Na/In/C composite anode

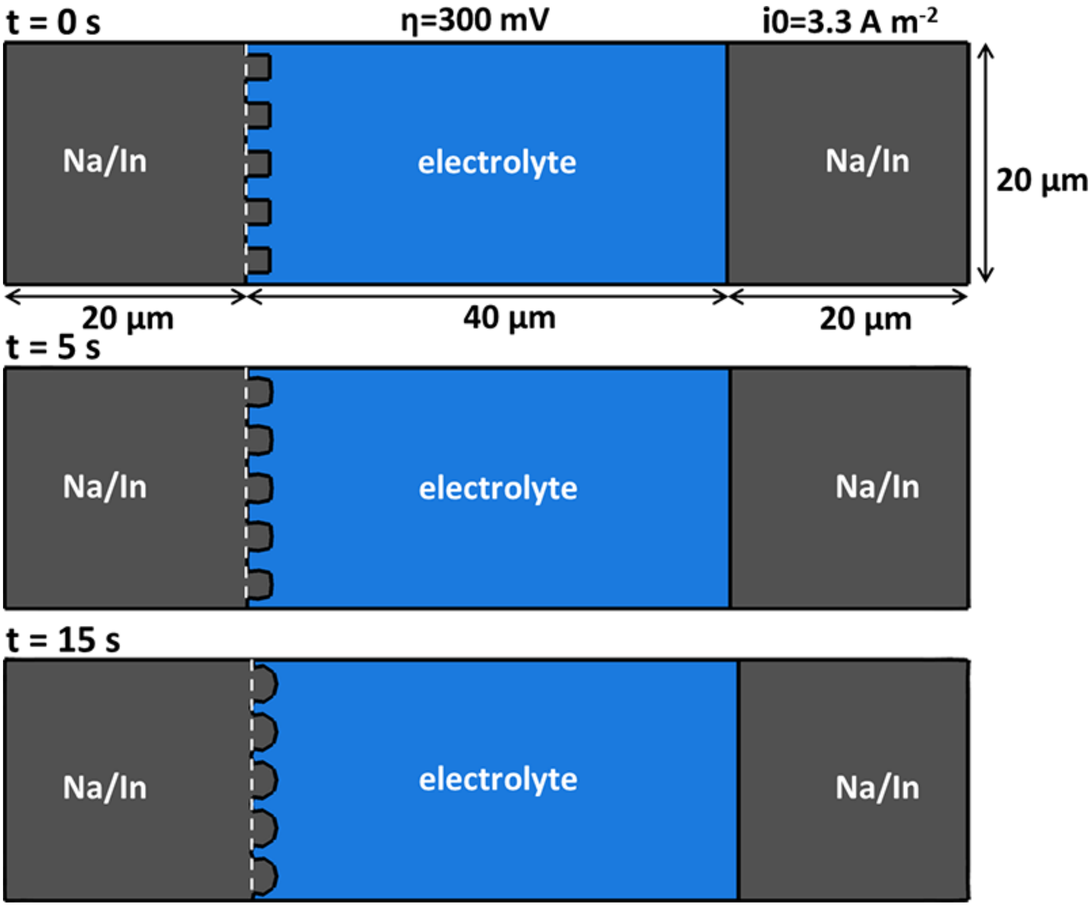

Fig. S22 Finite element simulations of Na/In electrode morphology evolution features with square-shaped bumps at various time (0 s, 5 s, 15 s) with a 300 meV overpotential and an exchange density of 3.3 A m^{-2}

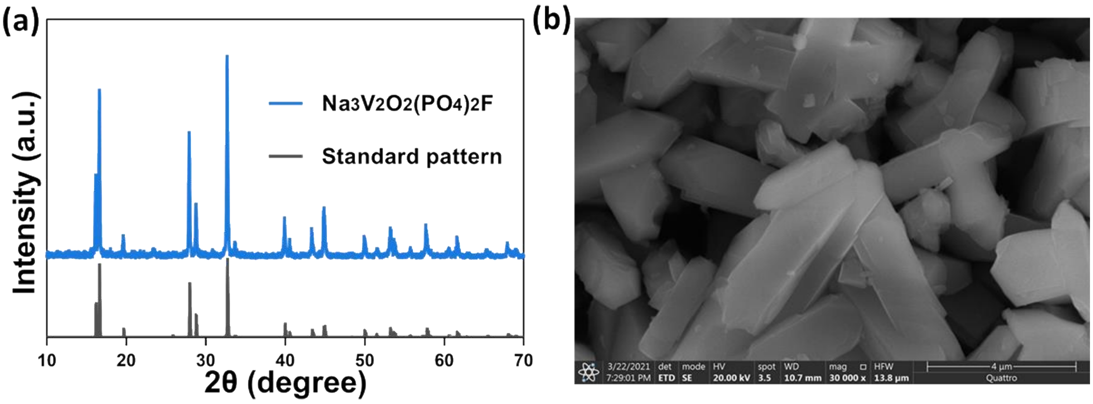

Fig. S23 (**a, b**) XRD pattern and SEM image of the as-synthesized $Na_3V_2O_2(PO_4)_2F$

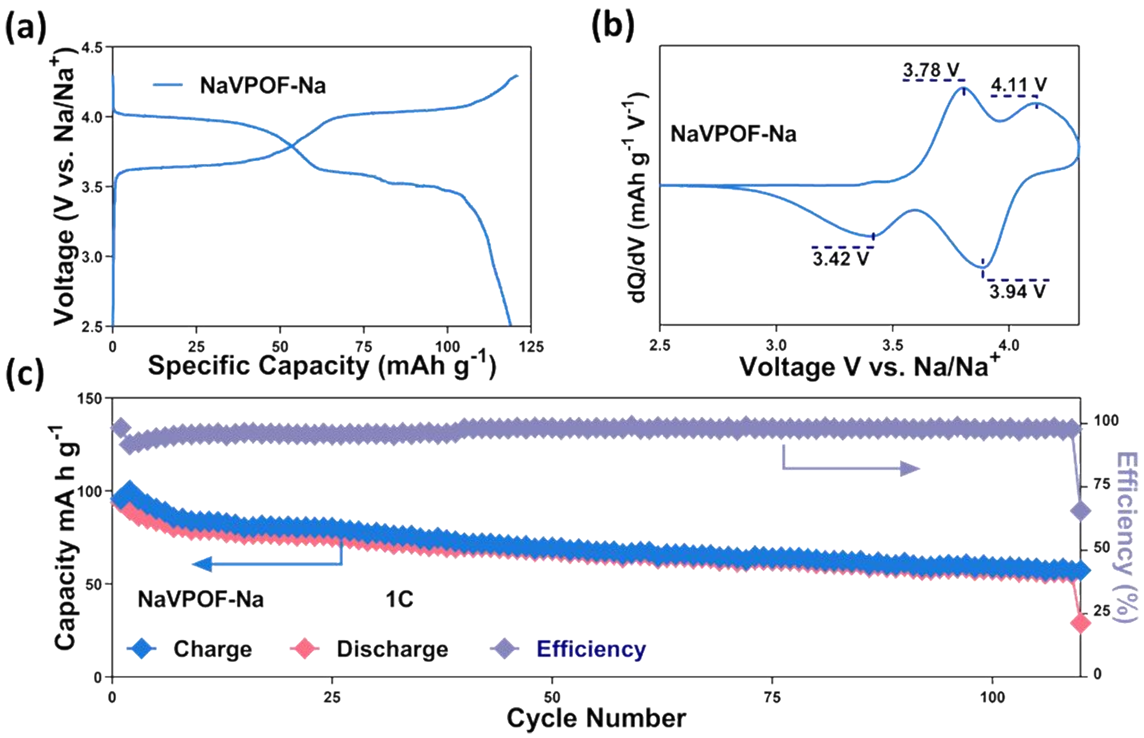

Fig. S24 Electrochemical evaluation of the sodium metal full batteries assembled NaVPOF cathode with the pure Na metal electrode: (**a**) voltage profile; (**b**) CV curve at a scan rate of 0.5 mV s^{-1}; (**c**) long-term cycling capability at 1 C within 2.5-4.3V
